# Cannot Ventilate: An Unexpected Cause of Respiratory Failure in a Ten-Year-Old Child

**DOI:** 10.7759/cureus.26965

**Published:** 2022-07-18

**Authors:** Zachary Mauro, Alexander Nguyen, Agata Dow

**Affiliations:** 1 Emergency Medicine, Henry Ford Health System, Wyandotte, USA

**Keywords:** difficult airway management, acute rheumatology, intubation response, pediatric respiratory failure, diffuse alveolar hemorrhage, pediatrics emergency, wegener's disease, intubation complication, pediatric respiratory diseases, granulomatosis with polyangiitis (gpa)

## Abstract

Granulomatosis with polyangiitis (GPA) is a necrotizing vasculitis known to affect the respiratory and renal systems. There are a multitude of clinical manifestations, many of which are not specific to the disease, such as dysfunction of the nasal, sinus, auditory, tracheal, pulmonary, ocular, renal, cardiac, and nervous systems. As a multisystemic illness without a "classic" presentation and insidious progression, it is often a challenging diagnosis. We report and discuss a case of a 10-year-old female with no significant past medical history who presented to the emergency department with a 10-day course of worsening respiratory symptoms. As her respiratory and clinical status began to precipitously decline, the decision was made to intubate the patient, which was performed without issue. Unfortunately, attempts at oxygenating and ventilating the patient were met with extreme resistance and difficulty-an airway situation that could have been catastrophic if not for quick reaction maneuvers performed that would ultimately go on to remedy the issue at hand. We hope to raise awareness regarding the airway challenges posed by GPA and delve into its management as a means of improving recognition and preparing clinicians to treat this condition.

## Introduction

Granulomatosis with polyangiitis (GPA) is a rare disease characterized by necrotizing vasculitis of small and medium-sized vessels plus granuloma formation. Clinical manifestations commonly involve both upper and lower respiratory tracts, as well as kidney dysfunction. Symptoms of nasal dysfunction, epistaxis, and pulmonary damage, characterized by infiltrates, effusions, and hemorrhage, are the most frequent. Although pulmonary involvement is the most common manifestation of GPA, diffuse pulmonary hemorrhage is a rare and potentially devastating sequela noted to be a cause of significant morbidity and mortality [1]. Prompt recognition and diagnosis of pulmonary hemorrhage are essential as it can lead to hypoxemia, acute respiratory distress syndrome, and eventually, respiratory failure requiring respiratory support [2]. There are few reports in the literature that describe challenges with endotracheal intubation secondary to airway stenosis and mucous plugs, and even fewer describe management in their presence. This is concerning as mucous plugs, present in regions of airway narrowing in the tracheobronchial regions, can lead to critical airway obstruction, which was highly suspected in our case [3]. While there is little published about airway management in the setting of GPA, there is even less reported about the diagnosis of GPA in children. Clinicians need to maintain a very high index of suspicion when caring for patients, especially children, with respiratory distress.

## Case presentation

A previously healthy 10-year-old African American female presented to a community hospital emergency department accompanied by her mother for worsening shortness of breath, epistaxis, and cough over the previous week. The child is up-to-date on her childhood vaccinations and has no medication allergies. There were also no reported familial health conditions at this time. She further reported that no one in the family is vaccinated for COVID-19 and that the child does attend in-person schooling. Her mother also added that the child has been experiencing intermittent epistaxis for the past 48 hours, which was managed with direct pressure and one episode of "crying blood," which resolved on its own.

Her vital signs were remarkable tachycardia with a heart rate of 136 beats per minute, hypertension with a blood pressure of 160/93, and tachypnea with a respiratory rate of 35 breaths per minute. The patient was afebrile with an oral temperature of 36.3 °C, and with a pulse oximetry reading of 93% on room air. The physical exam showed a well-nourished child with a weight of 56.1 kg, in obvious respiratory distress with audible wheezing and rales to the bilateral lung fields. Heart sounds lack a noticeable rub, murmur, or gallop. There was no epistaxis present and no dried blood noted in the nares.

Laboratory evaluation was remarkable for leukocytosis with bandemia, a white blood cell count noted at 19.1 K/µL, and an initial lactic acidosis of 2.4 mmol/L. Venous blood gas revealed a pH of 7.34 with a pCO_2_ of 56.1 mmHg and an HCO_3_ of 29.5 mmol/L. Electrolytes and renal function were all within normal limits. The COVID-19 polymerase chain reaction (PCR) was negative on two separate tests.

**Figure 1:**
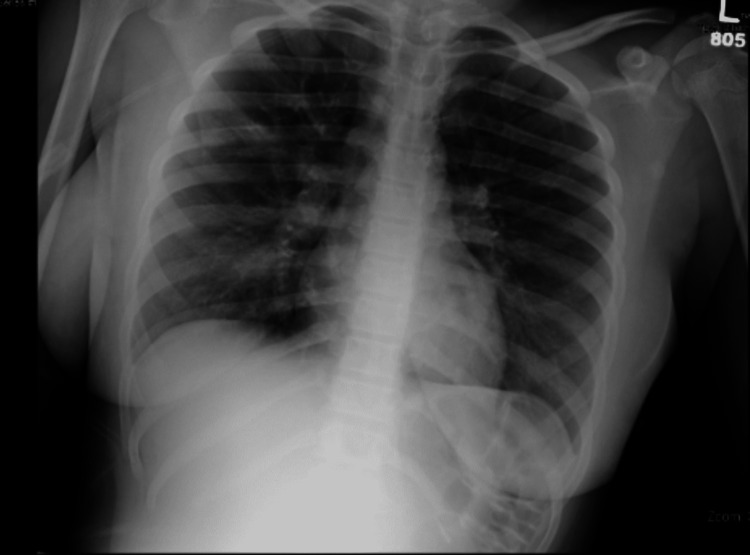
Chest X-ray demonstrating no acute intra-thoracic process.

**Figure 2:**
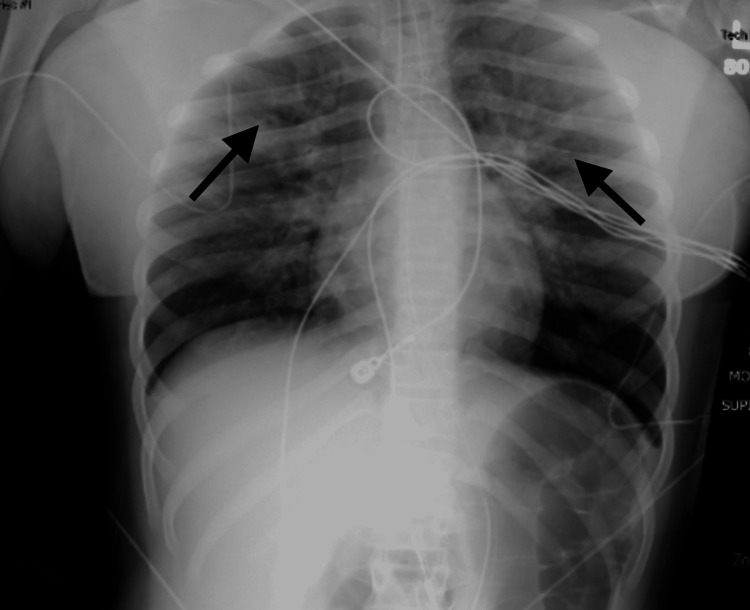
Chest X-ray with arrows demonstrating bilateral airspace opacities indicative of suspected fluid overload. Endotracheal tube in adequate position.

Initial management included intramuscular epinephrine, albuterol-ipratropium nebulizers, followed by a non-rebreather mask with high-flow oxygen, dexamethasone, and a 1-liter fluid bolus. Her initial chest X-ray was negative for pulmonary infiltrate, vascular congestion, and pneumothorax (Figure 1). Despite initial management, her condition worsened, with increased work of breathing, and a decision was made to intubate her due to impending respiratory failure. She developed apnea and desaturated down to 80%. Bag-valve-mask (BVM) ventilation was successful at correcting the hypoxia. She was induced with etomidate and rocuronium and was intubated utilizing video-laryngoscopy. The endotracheal tube (ETT) is visualized appropriately passing through the glottic opening. When ventilation was attempted using a bag valve mask, the respiratory therapist noted that the BVM could not easily be compressed and the child could not be ventilated. Due to concern that the ETT had become occluded or dislodged, the balloon was deflated and the patient was extubated. No occlusion was noted in the ETT at this time. Oxygen saturation at this time remained in the mid-90s and intubation was again performed utilizing video-laryngoscopy with BVM ventilation in-between attempts. On the second intubation attempt, the posterior oropharynx was without signs of trauma, bleeding, or occlusion. The ETT was again visualized passing through the glottic opening, the stylet was removed, and the cuff was inflated prior to the video-laryngoscopy blade being removed, ensuring ETT positioning. When ventilation was attempted, it was again noted that the BVM was exceptionally difficult to compress, and the child again could not be ventilated. Anesthesia was called emergently to the bedside. At this time, the patient was noted to become hypoxic with oximetry readings in the mid-70s. BVM ventilation was performed, now with only a slight improvement in oxygen saturations to the mid-80s. A third attempt at intubation was performed as before, again with failure to ventilate. At this time, the patient experienced worsening hypoxia and became bradycardic into his low 40s. Chest compressions were started, and the ETT was again removed in a similar fashion as before. During cardiopulmonary resuscitation, ventilation with BVM was noted to cause moderate gastric distention and no appreciable chest rise. The abdomen was manually decompressed without significant change in hemodynamics. The anesthesia team arrived at the bedside and successfully intubated the patient using video-laryngoscopy and a 7.0 ETT. It was at this time that ventilation was successful, with rapid improvement in oxygen saturation and heart rate. The total time of chest compressions was less than one minute with no pharmacology needed. Following intubation, bloody secretions were noted in the ETT, which were easily suctioned. Numerous blood clots and mucous plugs were also suctioned from the patient’s airway. Given concern for false-negative COVID-19 PCR, a repeat specimen was obtained, again with negative results. It is highly suspected that chest compressions and more forceful ventilation dislodged mucus plugs/blood clots in the airway, allowing for proper ventilation. An orogastric tube was placed for gastric decompression with the evacuation of non-bloody stomach contents. A repeat chest X-ray following intubation demonstrated adequate ETT positioning and bilateral diffuse patchy infiltrates with a formal read stating, "suspected to be due to fluid overload" (Figure 2). The patient was transferred to a tertiary children’s hospital for continued management via a pediatric critical care ambulance. She was admitted to the pediatric intensive care unit.

Cardiology was consulted due to evidence of pulmonary vascular congestion seen on chest X-ray and acquired an echocardiogram, which demonstrated normal ventricular function and cardiac anatomy, and ultimately signed off.

Infectious disease obtained blood cultures, respiratory syncytial virus/COVID-19/influenza PCR were all negative. The COVID-19 antigen was noted to be positive. A urinalysis was obtained due to the unknown source of the suspected infection. This was notable for microscopic hematuria and proteinuria with 3+ blood and 2+ protein. Urine, blood, and respiratory cultures had no growth after four days of incubation. She was treated with an abbreviated course of clindamycin, ceftriaxone, and vancomycin, discontinued due to negative cultures, and a full course of azithromycin for possible pneumonia.

Due to the patient requiring mechanical ventilatory support, repeat chest X-rays were obtained. Each of these new chest X-rays demonstrated continued evidence of bilateral infiltrates with areas of concerning worsening and increased infiltrate burden. It was at this time that the concern for pulmonary hemorrhages causing the infiltrates was seen. A renal ultrasound was obtained which demonstrated normal renal anatomy without evidence of hydronephrosis. During this ultrasound examination, there was evidence of a blood clot in the inferior vena cava.

A nephrologist was consulted due to the finding of microscopic hematuria, and a left renal biopsy was obtained due to concern about underlying vasculitis. Blood samples were remarkable at this time for elevated anti-proteinase 3 and c-antineutrophil cytoplasmic antibodies (ANCA) along with negative anti-double-stranded DNA, low anti-nuclear antibodies, and low C4 levels. Clinical symptoms along with biopsy and lab results were consistent with a diagnosis of c-ANCA-related vasculitis and glomerulonephritis with polyangiitis. The rheumatology service was consulted for the management of GPA and the patient was started on intravenous methylprednisolone and cyclophosphamide. Venous duplex ultrasounds were obtained of the upper and lower extremities, which demonstrated evidence of deep-vein-thrombosis in the right radial vein and cephalic vein at the antecubital fossa, for which the patient was started on enoxaparin.

The rheumatologist started the patient on a steroid burst with 1 gram of methylprednisolone followed by a slow, prolonged taper thereafter. The patient was also started on oral cyclophosphamide. The patient remained intubated for a period of 12 days and was then successfully extubated. She was able to complete prolonged continuous positive airway pressure (CPAP) trials starting on day 9.

She experienced a vast improvement in her clinical presentation, and following a prolonged hospital course, she was ultimately discharged home with cyclophosphamide, prednisone, and enoxaparin with continued outpatient rheumatology follow-up.

## Discussion

Granulomatosis with polyangiitis, previously known as Wegener’s disease (WD), is a vasculitis subtype affecting predominantly small vasculature. The disease is characterized by its association with ANCAs [3]. There is an incidence estimated at 3 per 100,000 individuals with a slight female predominance and is most often diagnosed in the 4th through 6th decades of life, with childhood diagnosis being significantly less common [4]. Initial presentation can take many forms and include constitutional, cutaneous, pulmonary, ear, nose and throat, and renal symptoms, with the most common presenting symptoms including constitutional manifestations [5]. Children typically present with more severe manifestations involving renal, gastrointestinal, and pulmonary organ involvement [6]. These can include airway obstruction and pulmonary hemorrhage, as seen in our patient.

The classic presentation includes pulmonary and renal involvement due to the abundance of capillaries surrounding the alveoli and glomeruli, respectively [7]. It is not uncommon for these patients, particularly children, to present with significant respiratory distress due to an obstructed airway from bleeding and clot formation within the airway itself [8]. Pulmonary hemorrhage can be suspected clinically and through basic radiology imaging and confirmed through direct visualization with bronchoscopy or advanced imaging such as CT or MRI. Hematuria is not always overt and may be missed unless clinical suspicion is high. Incidental findings of microscopic hematuria are common and may aid in the diagnosis and prompt further workup, such as in this patient who underwent a kidney biopsy.

The most striking moment in the emergency management of this patient revolved around the inability to ventilate after successful endotracheal intubation. When encountered with difficulties ventilating any patient, one should consider these four scenarios: dislodgement, obstruction, pneumothorax, or equipment failure.

In the case of this patient, there was low suspicion of a dislodged tube, as we had direct visualization of the endotracheal tube passing through the glottic opening. There was no evidence of pneumothorax on the initial chest X-ray and, given the lack of trauma, we had overall low suspicion. The patient was not attached to a ventilator, and the bag valve mask was fully functional, leading to a low suspicion of equipment failure. This left the high likelihood of an obstruction within the larger airway structures such as the trachea, bronchi, or potentially the endotracheal tube itself. Following the subsequent bradycardic cardiac arrest, with successful intubation, it was noted that multiple significantly sized blood clots were able to be suctioned from the patient's airway. This raised suspicion of pulmonary hemorrhage and obstructive blood clots as a cause of her respiratory distress and subsequent cardiopulmonary arrest.

In a child with otherwise normal anatomy who is noted to have a failure to ventilate, a subglottic obstruction of the larger airway structures should be considered. Remedying this situation may take many forms, including the use of in-line suction, advancing the endotracheal tube to attempt to dislodge the obstruction into a mainstem bronchus, or, in the case of cardiac arrest or the patient becoming unresponsive, chest compressions may provide dislodgement [9]. If the obstruction can not be dislodged or removed, a surgical cricothyroidotomy may be performed, but only if the obstruction is determined to be above the level of the cricothyroid membrane. In addition to subglottic obstructions, if there is a visualized supraglottic obstruction, removal with Magill forceps or Yankauer suction is indicated [9].

While granulomatosis with polyangiitis is an uncommon diagnosis for an emergency department provider, it should be considered when caring for an otherwise healthy child who presents with shortness of breath, epistaxis, and constitutional symptoms. As with every intubation, a failure to ventilate should result in a step-wise approach to troubleshooting the underlying cause.

## Conclusions

Shortness of breath continues to be a common complaint prompting evaluation in the emergency department and a leading chief complaint in the pediatric community. While the majority of patients will be diagnosed with a subtype of reactive airway disease and/or respiratory tract infection, the potential for an autoimmune cause should be considered, especially in those patients who do not respond to conventional treatment. In the case of this patient, there was no initial radiographic evidence of pulmonary infiltrate, and laboratory findings were not remarkable for infectious etiologies. The poor overall appearance and physical examination of the child did not match the severity of the laboratory and imaging results. Following intubation, any failure to ventilate should follow a stepwise, organized approach to diagnosing the underlying issue.

## References

[1] Garlapati P, Qurie A (2021). Granulomatosis With Polyangiitis - Statpearls - NCBI Bookshelf. 7 Dec.

[2] Han TS, Mahon RT (2006). Wegener's granulomatosis presenting with diffuse alveolar hemorrhage and negative antineutrophilic cytoplasmic antibody test. Mil Med.

[3] Masiak A, Struk-Panfill M, Zdrojewski Z (2015). Infectious complication or exacerbation of granulomatosis with polyangiitis?. Reumatologia.

[4] Bohm M, Gonzalez Fernandez MI, Ozen S (2014). Clinical features of childhood granulomatosis with polyangiitis (wegener's granulomatosis). Pediatr Rheumatol Online J.

[5] Robson JC, Grayson PC, Ponte C (2022). 2022 American College of Rheumatology/European Alliance of Associations for Rheumatology Classification Criteria for granulomatosis with polyangiitis. Arthritis Rheumatol.

[6] Cabral DA, Uribe AG, Benseler S (2009). Classification, presentation, and initial treatment of Wegener's granulomatosis in childhood. Arthritis Rheum.

[7] James KE, Xiao R, Merkel PA, Weiss PF (2017). Clinical course and outcomes of childhood-onset granulomatosis with polyangiitis. Clin Exp Rheumatol.

[8] Lee PY, Adil EA, Irace AL (2017). The presentation and management of granulomatosis with polyangiitis (Wegener's Granulomatosis) in the pediatric airway. Laryngoscope.

[9] Clinical Practice Guidelines - Foreign Bodies Inhaled .” The Royal Children's Hospital Melbourne, Mar Mar (2022). Foreign bodies inhaled. https://www.rch.org.au/clinicalguide/guideline_index/Foreign_bodies_inhaled/.

